# Characterization and Mutational Analysis of Omega-Class GST (*GSTO1*) from *Apis cerana cerana*, a Gene Involved in Response to Oxidative Stress

**DOI:** 10.1371/journal.pone.0093100

**Published:** 2014-03-25

**Authors:** Fei Meng, Yuanying Zhang, Feng Liu, Xingqi Guo, Baohua Xu

**Affiliations:** 1 College of Life Sciences, Shandong Agricultural University, Taian, Shandong, P. R. China; 2 College of Animal Science and Technology, Shandong Agricultural University, Taian, Shandong, P. R. China; Instituto de Biociencias - Universidade de São Paulo, Brazil

## Abstract

The Omega-class of GSTs (GSTOs) is a class of cytosolic GSTs that have specific structural and functional characteristics that differ from those of other GST groups. In this study, we demonstrated the involvement of the *GSTO1* gene from *A. cerana cerana* in the oxidative stress response and further investigated the effects of three cysteine residues of GSTO1 protein on this response. Real-time quantitative PCR (qPCR) showed that *AccGSTO1* was highly expressed in larvae and foragers, primarily in the midgut, epidermis, and flight muscles. The *AccGSTO1* mRNA was significantly induced by cold and heat at 1 h and 3 h. The TBA (2-Thiobarbituric acid) method indicated that cold or heat resulted in MDA accumulation, but silencing of *AccGSTO1* by RNAi in honeybees increased the concentration of MDA. RNAi also increased the temperature sensitivity of honeybees and markedly reduced their survival. Disc diffusion assay indicated that overexpression of AccGSTO1 in *E. coli* caused the resistance to long-term oxidative stress. Furthermore, AccGSTO1 was active in an *in vitro* DNA protection assay. Mutations in Cys-28, Cys-70, and Cys-124 affected the catalytic activity and antioxidant activity of AccGSTO1. The predicted three-dimensional structure of AccGSTO1 was also influenced by the replacement of these cysteine residues. These findings suggest that *AccGSTO1* plays a protective role in the response to oxidative stress.

## Introduction

Reactive oxygen species (ROS), such as oxygen radical superoxide (O_2_^–^) and hydroxyl (OH^–^), are constantly generated during aerobic metabolism. Although the rate of oxidant scavenging maintains a dynamic balance under normal conditions, an imbalance is created when overproduction of endogenous or exogenous oxidants exceeds the intracellular antioxidant capacity, leading to excessive ROS and oxidative stress [1], [2]. As there are varied levels of ROS in living organisms, ROS can have both beneficial and harmful effects on a diverse array of biological processes. In insects, there is compelling evidence that ROS can regulate ageing. In other words, oxidative stresses can decrease their life span [3]. ROS can also act as modulators of signal transduction pathways [4], [5] or can accelerate the aggregation of abnormal proteins, inducing oxidative stress that is associated with many diseases such as atherosclerosis, Alzheimer's disease, and Parkinson's disease, all of which are connected to ageing and life span [6], [7]. Thus, to defend against oxidative damage, aerobic organisms have evolved autologous enzymatic antioxidant systems, including primary and secondary antioxidant enzymes [8].

The glutathione S-transferases (GSTs; EC 2.5.1.18) are a family of phase II enzymes that are widely found in both eukaryotic and prokaryotic cells. A prominent characteristic of these proteins is the ability to utilize glutathione in reactions, contributing to the biotransformation and degradation of various environmental xenobiotics, such as drugs, insecticides, and intracellular ROS [9], [10]. Unlike mammalian GSTs, insect GSTs can be grouped into six cytosolic classes: Delta (GSTD), Epsilon (GSTE), Omega (GSTO), Sigma (GSTS), Theta (GSTT), Zeta (GSTZ), one structurally unrelated microsomal class (GSTmic), and one unclassified class (u) [8], [11], [12]. Although some GSTs have a canonical GST structure and exhibit overlapping substrate specificities, other members are highly specific.

The Omega-class GSTs have unique structural and functional characteristics that differ from the other GST groups. The first GSTO was found through a bioinformatic analysis of human expressed sequence tags [13]. Subsequently, GSTOs have been identified in plants [14], yeast [15], insects [16], and bacteria [17], and the number of GSTO genes varies depending on the species. For instance, three Omega GST genes have been found in the human genome [18], two GSTO genes have been found in mice and rats [11], and two GSTO genes have been identified in honeybees [8]. These GSTOs also display distinct genetic organizations, crystal structures, substrate specificities, and catalytic activities [19].

In HsGSTO1, a novel cysteine residue (Cys-32) is located in the active site, an arrangement that is distinct from the prototypical serine and tyrosine residues in the other classes of the GST superfamily [13], [20]. The crystal structure of HsGSTO1 revealed that Cys-32 can form a mixed disulfide bond with the thiol of GSH precisely over the helix axis at the N-terminal region [13]. GSTO1 exhibits a canonical cytosolic GST fold, however, it has unique N-terminal and C-terminal extensions that are not observed in the other GST classes. These two domains of GSTO1 interact to form a distinct structural unit that is crucial for substrate specificity [20]. It is therefore not surprising that GSTO1 has unique enzymatic properties, most significantly thiol transferase (TTase) and dehydroascorbate reductase (DHAR) activities [22], [23]. Activities against 1-chloro-2,4-dinitrobenzene (CDNB) or ethacrynic acid (ECA), typical substrates for other classes of GSTs, are low or not detected in GSTO1 [23], [24].

In early studies, elevated GSTO1 activity was associated with resistance to radiation and cytotoxic drugs [25], and the participation of these enzymes in the biomethylation pathway of inorganic arsenic metabolism was later reported [26]. Recent evidence suggests that GSTO1 is involved in protection against oxidative stress, including platinum resistance, the heat shock response, and the response to UV-B light [23], [24], [27], [28]. The *GSTO1* gene also modifies the age-at-onset of Alzheimer's disease and Parkinson's disease [7]. Other proposed roles of GSTO1 include the modulation of Ca^2+^ ion channels [29], the participation in IL-1β activation [30], and a direct interaction in pyrimidodiazepine synthesis [31].

The activity and function of GSTO1 can be influenced by alterations in the active site. Mutation of the active site Cys-32 residue in human GSTO1 causes a strong increase in activity toward CDNB [22], whereas the Asp-140 variant has a lower thiol transferase activity [32]. The replacement of Cys-33 of GSTO1 in *Caenorhabditis elegans* elevates the worm's sensitivity to cumene hydroperoxide [23], and mutations of Cys-38 and Pro-39 in silkmoths also affect BmGSTO1 activity [24].

The Asian honeybee (*Apis cerana cerana*) is a valuable indigenous species that plays an important role in the pollination of flowering plants, especially in low temperature environments. In recent decades, global warming and excessive pesticide use have threatened the existence of this species. Although some GST genes have been identified in *A. cerana cerana*, the specific GSTO1 remains unknown. Furthermore, the influence of different GSTO1 cysteine residues on the oxidative stress response has not yet been studied. Thus, to further elucidate the protective role of GSTO1 in a multicellular organism, we isolated and characterized a *GSTO1* gene from *A. cerana cerana*, and investigated the biochemical properties and antioxidant capacity of AccGSTO1; three mutated proteins were also investigated. Our findings provide valuable insight into the function of GSTO1 in the oxidative stress response.

## Experimental Procedures

### Insects, tissue dissection and stress treatment

Colonies of *A. cerana cerana* were reared in beehives at the experimental apiary of Shandong Agricultural University, Taian, China. Each larval and pupal stage was identified by calculating the number of days after the queen laid eggs. Adult workers were collected at the correct time after marking the newly emerged bees with paint. The brain, epidermis, flight muscle, midgut, and mandibular glands were dissected from the adult workers on ice and stored at −80°C. For the stress treatments, the adult workers were fed water and pollen and were exposed to 75% relative humidity at different temperatures: 4°C, 15°C, 25°C, 34°C, 43°C, and 50°C. Thereafter, the treated bees were frozen in liquid nitrogen and stored at −80°C until use.

### RNA extraction, cDNA synthesis, and DNA isolation

Total RNA was rapidly extracted from the dissected tissues using the TRIzol reagent (Invitrogen, Carlsbad, CA, USA) according to the manufacturer's instructions. After digestion with RNase-free DNase I, the first-strand cDNA was synthesized using the EasyScript First-Strand cDNA Synthesis SuperMix (TransGen Biotech, Beijing, China). Genomic DNA was isolated using the EasyPure Genomic DNA Extraction Kit (TransGen Biotech, Beijing, China) in accordance with the manufacturer's protocol.

### Cloning of the *AccGSTO1* gene

The procedures were performed as previously described [33]. Briefly, conserved primers GP1 and GP2 were designed to obtain an internal fragment of the *AccGSTO1* gene by PCR. RACE primers and full-length primers were subsequently generated to verify the *AccGSTO1* cDNA sequence. Several specific primers were used to isolate the *AccGSTO1* genomic DNA. All primers and their sequences are listed in Table S1.

### Real-time quantitative PCR

Real-time quantitative PCR (qPCR) was performed using the SYBR PrimeScript™ RT-PCR Kit (TaKaRa, Dalian, China) in a 25-μL reaction volume using a CFX96TM Real-Time PCR Detection System. The β-actin gene (GenBank ID: XM640276) was selected as a reference gene due to its stable expression in honeybees [34]. The following amplification reaction protocol was used: pre-denaturation at 95°C for 30 s; 40 cycles of denaturation at 95°C for 5 s, annealing at 56°C for 15 s, and extension at 72°C for 15 s; and a single melt cycle from 65°C to 95°C. All the conditions were analyzed in three independent biological replicates, and the PCR reactions were performed in triplicate. The data were analyzed using the CFX Manager software, version 1.1, with the 2−ΔΔCt method. The statistical significance was determined by a Duncan's multiple range test using Statistical Analysis System (SAS) version 9.1 software.

### Determination of the malonyldialdehyde (MDA) concentration

Lipid oxidation results in the generation of many harmful secondary products and MDA can act as a reliable indicator of lipid peroxidation to indirectly reflect cellular damage. In this assay, the total proteins from the foragers held at different temperatures were rapidly extracted using a Tissue Protein Extraction Kit (CWBIO, Beijing, China). Each sample was digested with 990 μl of tissue protein extraction reagent with 10 μl of PMSF and incubated at 4°C for 20 min, followed by centrifugation at 10,000 g for 10 min. The supernatant was removed and diluted at a 1∶10 ratio (v/v). The MDA concentration was measured using an MDA assay kits (Institute of Biological Technology of Jiancheng, Nanjing, PR China). Because TBA was used as the substrate, this method was also called the TBA method. Simply, TBA combined with MDA to form red products, and the absorbance was measured at 532 nm. The MDA concentration was expressed as nmol of MDA per mg of protein. Three replicates were performed in each experiment.

### RNA-mediated interference (RNAi) of *AccGSTO1*

Double-stranded RNAs (dsRNAs) were synthesized as described in an established method [33]. Briefly, specific primers with a T7 polymerase promoter were produced to synthesize the ds*AccGSTO1* using the Ribo Max™^ T7 ^Large Scale RNA Production Systems (Promega, Madison, WI, USA). After digestion with DNase I and isopropanol extraction, the dsRNA was annealed by gradual cooling from 95°C to room temperature. dsGFP was selected as a control [35]. All dsRNAs were transported by oral feeding into the foragers.

### Stress resistance assays

The foragers were collected from source colonies and divided into nine groups (n = 80/group). Groups 1–3 of the foragers were fed a normal diet. For Groups 4–6 and Groups 7–9, double-stranded *GFP* or *AccGSTO1* (20 μg) were added to the normal diet, respectively. All groups were maintained in incubators at a constant temperature (50°C) and humidity (75%) for 0.5–5 h after 1 day. The survival rates of the foragers were evaluated hourly. A forager was scored as dead when it did not respond to any stimulus.

### Overexpression and purification of recombinant proteins

The full-length *AccGSTO1* cDNA was integrated into the prokaryotic expression vector pET-30a (+) and subsequently transformed into *Escherichia coli* BL21 (DE3) cells. After the cells reached an optical density (OD)_600_ of 0.2–0.5, isopropyl-*β*-D-thiogalactopyranoside (IPTG) was added to the culture at a final concentration of 0.6 mM to induce protein expression. SDS-PAGE was used to analyze the homogeneity of the proteins. The protein purification was performed as previously described [36]. Briefly, cell pellets were resuspended in lysis buffer (phosphate-buffered saline containing 15% glycerol, 0.3 M NaCl, and 20 mM imidazole, pH 7.4). After sonication and centrifugation, the supernatant was applied to a 1-mL HisTrap™ FF column (GE Healthcare, Uppsala, Sweden) for further purification. The proteins were extensively eluted with elution buffer (phosphate-buffered saline containing 0.25 M imidazole, pH 7.4 and 0.5 M arginine). The protein concentration was determined using Coomassie Brilliant Blue G-250, and bovine serum albumin was used as a standard.

### Antibody preparation and immunoblot analysis

Purified AccGSTO1 protein (100 μg/ml) mixed with Freund's complete adjuvant was subcutaneously injected into six white male mice. A week later, the mice were again injected with a mixture of 25 μg/ml purified protein and Freund's incomplete adjuvant for increased immunity. In all, the immunization was performed three times at one-week intervals. Three days after the last injection, the antisera were collected by centrifugation at 3000 rpm for 15 min and stored at −80°C [37]. The total proteins from the foragers were rapidly extracted using a Tissue Protein Extraction Kit (CWBIO, Beijing, China). Purified AccGSTO1 and equal amounts of total proteins were subjected to 12% SDS-PAGE and then electrotransferred to a polyvinylidene difluoride membrane (PVDF, Millipore Corporation, UK). The immunoblot was performed using the anti-AccGSTO1 polyclonal antibody (1∶100 dilution) and peroxidase-conjugated goat anti-mouse secondary antibody (Dingguo, Beijing, China) at a dilution of 1∶2000 (v/v). Subsequently, the immunoblot was visualized using the SuperSignal West Pico Trial Kit (Thermo Scientific Pierce, IL, USA).

### Site-directed mutagenesis

Three mutants (C28A, C70A, and C124A) were generated to replace each of the three cysteine residues in AccGSTO1. PCR-based mutagenesis was performed using the Quick-Change Site-Directed Mutagenesis Kit (Agilent Technologies, Wilmington, DE, USA) according to the manufacturer's recommendations. The recombinant plasmid containing *AccGSTO1* was used as the template, and nucleotide sequencing was performed to verify the mutations. The mutants were then transformed into DE3 cells for protein expression.

### Disc diffusion assay

The *E. coli* cultures containing the target protein were plated on LB-kanamycin agar plates and incubated at 37°C for 1 h. Sterile filter discs (6-mm diameter) were saturated with 20 μL aliquots of different concentrations of compounds, including paraquat (0, 50, 100, 200, and 300 mM), cumene hydroperoxide (0, 25, 50, 75, and 100 mM) and tert-butyl hydroperoxide (0, 15, 20, 25, and 30 mM). All the discs were placed on the surface of the agar and incubated at 37°C for 24 h. The statistical significance of the inhibition zone was calculated using SAS version 9.1 software.

### DNA cleavage assay with the MFO system

Reaction mixtures of 25 μL, including 100 mM HEPES buffer, 3 mM FeCl_3_, 10 mM DTT, and increasing concentrations of protein in equal volumes, were incubated at 37°C for 30 min. Subsequently, 300 ng of supercoiled pUC19 plasmid DNA was added to the reaction mixture, which was incubated for 2.5 h at 37°C. Agarose gel electrophoresis was performed using a 1% gel to determine DNA cleavage. The signal intensities of the bands were determined using the Quantity-One^-^™ Image Analysis software (Bio-Rad, Hercules, CA, USA).

### Measurements of enzyme activity

The enzymatic activity toward 1-chloro-2,4-dinitrobenzene (CDNB) was measured using spectrophotometry, as described by Habig [38]. The glutathione-dependent dehydroascorbate reductase (DHAR) activity was determined spectrophotometrically with DHA at 265 nm, as described by Wells [39]. The glutathione peroxidase activity was measured by recording at 340 nm the oxidation of NADPH in a reaction mixture containing NADPH, GSH, glutathione reductase and H_2_O_2_ [40]. Alternatively, cumene hydroperoxide or t-butylhydroperoxide was also used instead of H_2_O_2_ in the reaction to detect peroxidase activity.

### Optimum temperature and pH and kinetic analysis

The effects of pH and temperature on enzymatic activity were examined using a standard DHAR assay, as described previously [36]. To determine the optimal temperature, the reaction was initiated by adding DHA to the protein mixture over a temperature range of 5°C to 55°C and recording for 2 min. Citrate buffer (50 mM, pH 4.0–5.0), sodium phosphate buffer (50 mM, pH 6.0–7.0), and Tris-HCl buffer (50 mM, pH 8.0–9.0) were used to determine the optimal pH. The apparent kinetic parameters of the recombinant proteins were determined using a GSH range of 0.2–4.0 mM and a fixed DHA concentration of 1 mM. Similarly, the apparent Km and Vmax values for DHA were determined using a DHA range of 0.1–2.0 mM and a fixed GSH concentration of 2.25 mM. All of the reactions were measured at pH 8.0 and 25°C. The Michaelis constant was calculated using the Lineweaver-Burk method in the Hyper program [41].

### Bioinformatic analysis

The amino acid sequence was aligned with homologous GSTO1 sequences using DNAman version 5.2.2 (Lynnon Biosoft, Quebec, Canada). The conserved domains in GSTO1 were predicted using the NCBI server (http://www.ncbi.nlm.nih.gov/Structure/cdd/cdd.shtml). A phylogenetic analysis was performed using the neighbor-joining method with the Molecular Evolutionary Genetic Analysis (MEGA) software version 4.0. All the tertiary structures were drawn using Swiss-PdbViewer.

## Results

### Identification and sequence analysis of *AccGSTO1*

**Figure 1:**
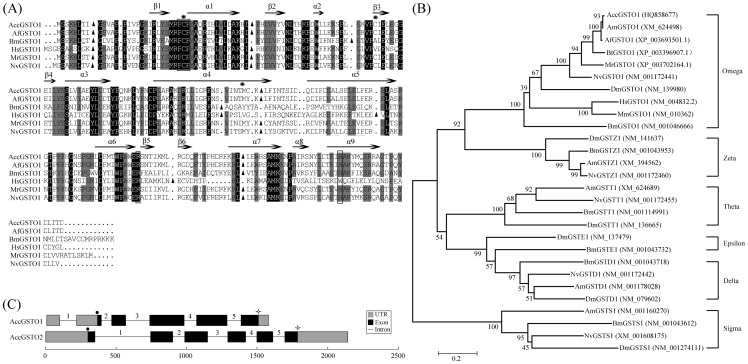
Sequence analysis of the AccGSTO1. (A) Alignment of AccGSTO1 with the Omega-class GSTs from *Apis florea* (AfGSTO1), *Bombyx mori* (BmGSTO1), *Homo sapiens* (HsGSTO1), *Megachile rotundata* (MrGSTO1) and *Nasonia vitripennis* (NvGSTO1). Identical amino acids are shaded in black and similar amino acids are shown in gray. The putative secondary structures are displayed, and the H-site regions are boxed. The Cys residues in AccGSTO1 are marked by asterisks. The intron/exon junctions are indicated by triangles. (B) Phylogenetic relationships among GSTO1 and other GST families. Six main groups are shown. Acc: *Apis cerana cerana*; Af: *Apis florea*; Am: *Apis mellifera*; Bm: *Bombyx mori*; Bt: *Bombus terrestris*; Dm: *Drosophila melanogaster*; Hs: *Homo sapiens*; Mm: *Mus musculus*; Mr: *Megachile rotundata*; Nv: *Nasonia vitripennis*. (C) Comparison between the genomic structures of *AccGSTO1* and *AccGSTO2*. The translational initiation codons (ATG) and termination codons (TAA) are marked with dots and asterisks, respectively.

The cDNA sequence of *AccGSTO1* (GenBank ID: KF496073) is 1,040 bp long and is composed of a 246-bp 5′ untranslated region (UTR), a 68-bp 3′ UTR, and a 726-bp open reading frame (ORF) that encodes a peptide with a predicted molecular mass of 27.9 kDa. A multiple sequence alignment revealed that the AccGSTO1 N-terminal region is highly conserved with other species (Fig. 1A). Residues that contribute to the H-site region, as described for HsGSTO1 [13], were either conserved or conservatively replaced. However, unlike HsGSTO1, AccGSTO1 contains two additional Cys residues, and the active site Cys-28 is not predicted to be located in the α1 helix. Furthermore, a neighbor-joining phylogenetic tree including proteins from the six cytosolic GST superfamilies of different species was generated (Fig. 1B). The tree clearly grouped the Omega family in a cluster distinct from the other five classes of GSTs. Compared to *Bombus terrestris* and *Nasonia vitripennis*, GSTO1 from *A. cerana cerana* was placed closest to *Apis mellifera* and *Apis florea*. The genomic structure of *AccGSTO1* was also analyzed and found to be 1,579 bp (GenBank ID: KF496074), containing five introns. In contrast, *AccGSTO2* (GenBank ID: JX456219) is much larger than *AccGSTO1*, encompassing 2,147 bp [36]. Although there are five introns in both *AccGSTO* genes, the first intron of *AccGSTO1* is located in the 5′ UTR (Fig. 1C).

### Expression profiles of *AccGSTO1*

**Figure 2:**
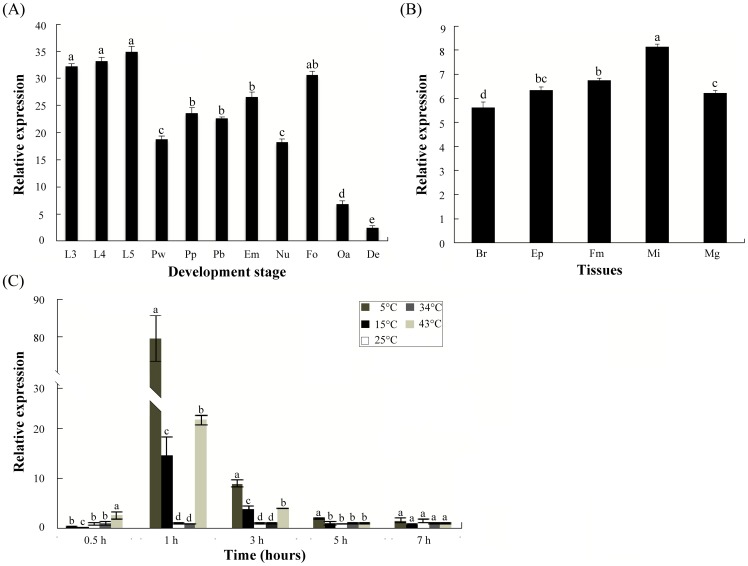
The expression profile of *AccGSTO1* determined using qPCR. (A) *AccGSTO1* mRNA expression at different developmental stages. Total RNA was isolated from larvae (L3-third instars, L4-fourth instars, and L5-fifth instars), pupae (Pw-white eyes pupae, Pp-pink eyes pupae, and Pb-brown eyes pupae), and adults (Em-newly emerged bees, Nu-nurse bees, Fo-foragers, Oa-old bees, and De-dead bees). (B) The tissue distribution of *AccGSTO1* expression. Total RNA was extracted from dissected brain (Br), epidermis (EP), flight muscle (Fm), midgut (Mi), and mandibular gland (Mg) tissues of foragers. (C) Expression of *AccGSTO1* after different temperature treatments (4°C, 15°C, 25°C, 34°C, and 43°C). Samples were collected at 0.5, 1, 3, 5, and 7 h. Bees at 34°C were used as a control. The vertical bars represent the mean ± S.E.M. (n = 3). The different letters above the columns indicate significant differences (P<0.05) according to Duncan's multiple range test performed using SAS version 9.1 software.

Stage-specific expression profiling showed that the highest expression levels were detected in larvae and foragers. The amount of *AccGSTO1* mRNA decreased rapidly in older bees, and almost no significant expression was detected in dead bees that died from natural causes (Fig. 2A). The tissue-specific expression analysis showed that *AccGSTO1* mRNA was expressed in all the tissues tested, with higher expression in the midgut, epidermis, and flight muscles and a relatively low level in the brain (Fig. 2B). Conversely, the highest expression of *AccGSTO2* has been detected in the brain [36]. As shown in Fig. 2C, after different temperature treatments, the expression of *AccGSTO1* was significantly induced at 4°C, 15°C, and 43°C compared with the expression level at 34°C (P <0.01). The highest expression of *AccGSTO1* was detected after 1 h of temperature treatment. The overall expression profiles of *AccGSTO1* were different from those previously obtained for *AccGSTO2*, but cold and heat treatment have also been shown to induce AccGSTO2 in a similar way [36]. These results reveal that the expression of *AccGSTO1* may be linked to the life span of the honeybee and the response to abiotic stress.

### MDA content during temperature-induced oxidative stress

**Figure 3:**
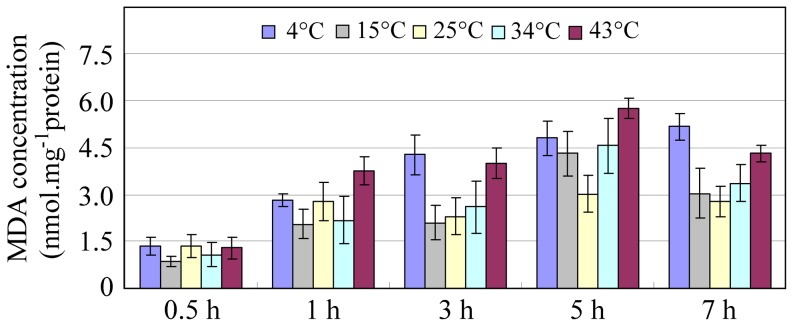
The detection of the MDA content at different temperatures. In all experiments, foragers at 34°C were used as the controls. The effects of temperature-induced oxidative stress on the MDA concentration (nmol mg^−1^ protein) in foragers. The vertical bars represent the mean ± S.E.M. (n = 3).

To determine the influence of the temperature on oxidative stress, MDA concentration (nmol/mg protein) was measured *in vitro* by the TBA method. As shown in Fig. 3, 4°C (cold) and 43°C (heat) resulted in a higher accumulation of lipid peroxides compared with the other temperatures. In general, the MDA concentration gradually increased from 0.5 h to 7 h, suggesting that many harmful secondary products were continuously generated. The high expression of *AccGSTO1* observed after 1 h and 3 h of cold or heat stress indicates that *AccGSTO1* may play a role in antioxidant defense when MDA levels are rising.

**Figure 4:**
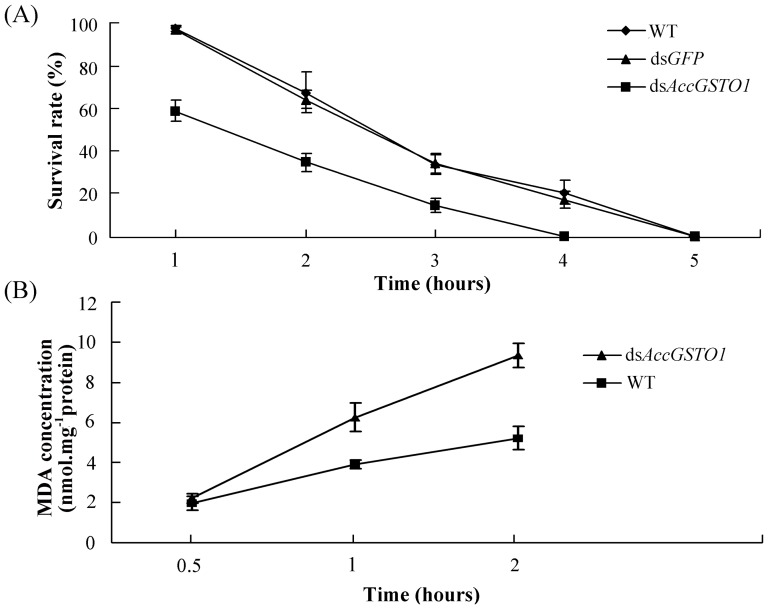
The effects of the RNAi-mediated silencing of AccGSTO1. (A) The survival rate of *AccGSTO1* RNAi-treated foragers compared with controls at 50°C. The foragers were collected and divided into nine groups (n = 80/group). Groups 1-3 and Groups 4-6 were used as the controls and were fed a normal diet or a normal diet supplemented with ds*GFP*, respectively. Groups 7–9 were fed ds*AccGSTO1*. All the foragers were placed in incubators at a constant temperature (50°C) and humidity (75%) for 1–5 h. (B) The MDA content of RNAi-treated foragers compared with controls at 50°C. All the foragers were placed in incubators at a constant temperature (50°C) and humidity (75%) for 0.5–2 h.

### RNAi-mediated silencing of *AccGSTO1* leads to temperature sensitivity

Colder temperatures induced the foragers to enter a sleeping stage, making it impossible to accurately determine their survival. Therefore, the foragers were fed double-stranded *AccGSTO1* RNA and were placed at higher temperatures (50°C) to measure their survival rate; foragers fed a normal diet and ds*GFP* were placed under the same conditions to be used as controls. As shown in Fig. 4A, the survival rate of the *AccGSTO1* RNAi foragers was significantly lower than that of the control foragers, and there were no obvious signs of life in the foragers after 4 h at 50°C. In addition, RNAi-mediated inhibition of *AccGSTO1* also induced a higher MDA concentration after 1 h and 2 h at 50°C (Fig. 4B). These results indicate that the RNAi-mediated silencing of *AccGSTO1* increases the temperature sensitivity of foragers.

### Characterization of the recombinant proteins

**Figure 5:**
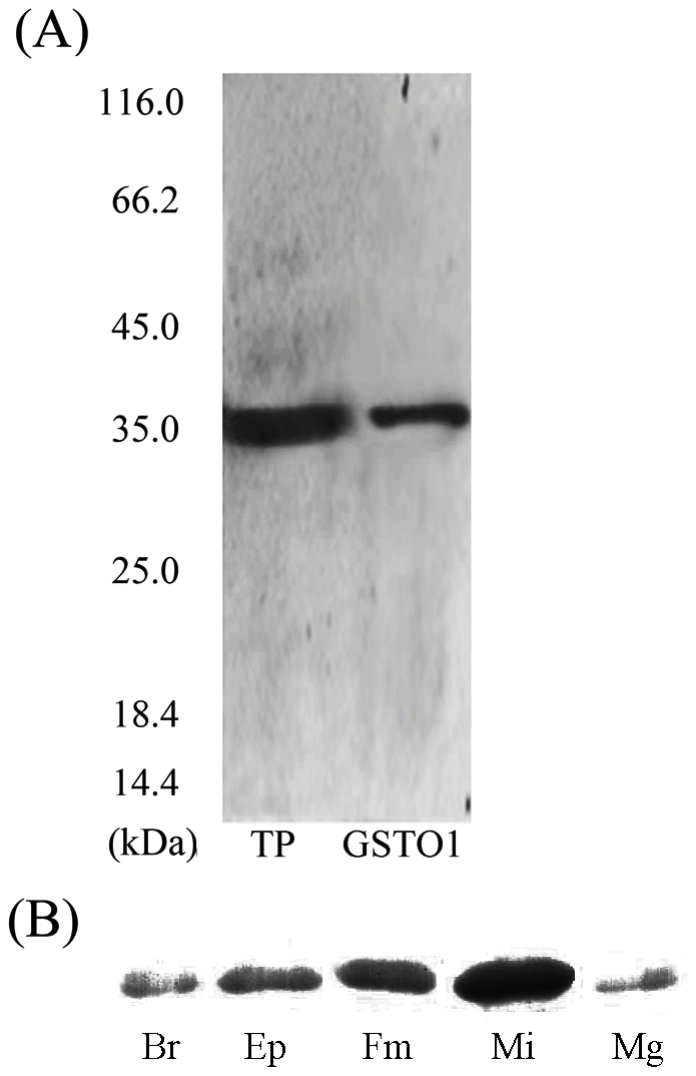
Immunoblot analysis of AccGSTO1 (A) Immunoblot analysis was performed on total protein from foragers (TP) and purified AccGSTO1. (B) Immunoblot analysis was performed with different tissues from the foragers. Each lane was loaded with an equivalent amount of protein. Br: brain, EP: epidermis, Fm: flight muscle, Mi: midgut, Mg: mandibular gland.

To determine whether the different Cys residues critically influence the antioxidant functions of AccGSTO1, each residue was mutated to Ala using site-directed mutagenesis. The three mutants were named C28A, C70A, and C124A and were overexpressed in *E. coli* BL21 (DE3) cells as recombinant proteins. Although the predicted size of the AccGSTO1 monomer and the three mutants was 27.9 kDa, the proteins with a His-tag migrated at approximately 35 kDa during sodium dodecyl sulfate polyacrylamide gel electrophoresis (SDS-PAGE). Further purification was performed using HisTrap™ FF columns (Figure S1). These results demonstrate that the wild-type and three mutant versions of AccGSTO1 were successfully overexpressed. Moreover, polyclonal antibodies against AccGSTO1 were raised in white male mice following standard immunization procedures. Immunoblotting showed that AccGSTO1 was obviously detected in both the total proteins of the foragers and the purified AccGSTO1 (Fig. 5A). Moreover, the expression of AccGSTO1 protein in different tissue was consistent with the mRNA expression levels (Fig. 2B, Fig. 5B). These results indicate that the anti-AccGSTO1 antibody is reasonably specific.

### Effects of the C28A, C70A, and C124A mutations on the oxidative stress response

**Figure 6:**
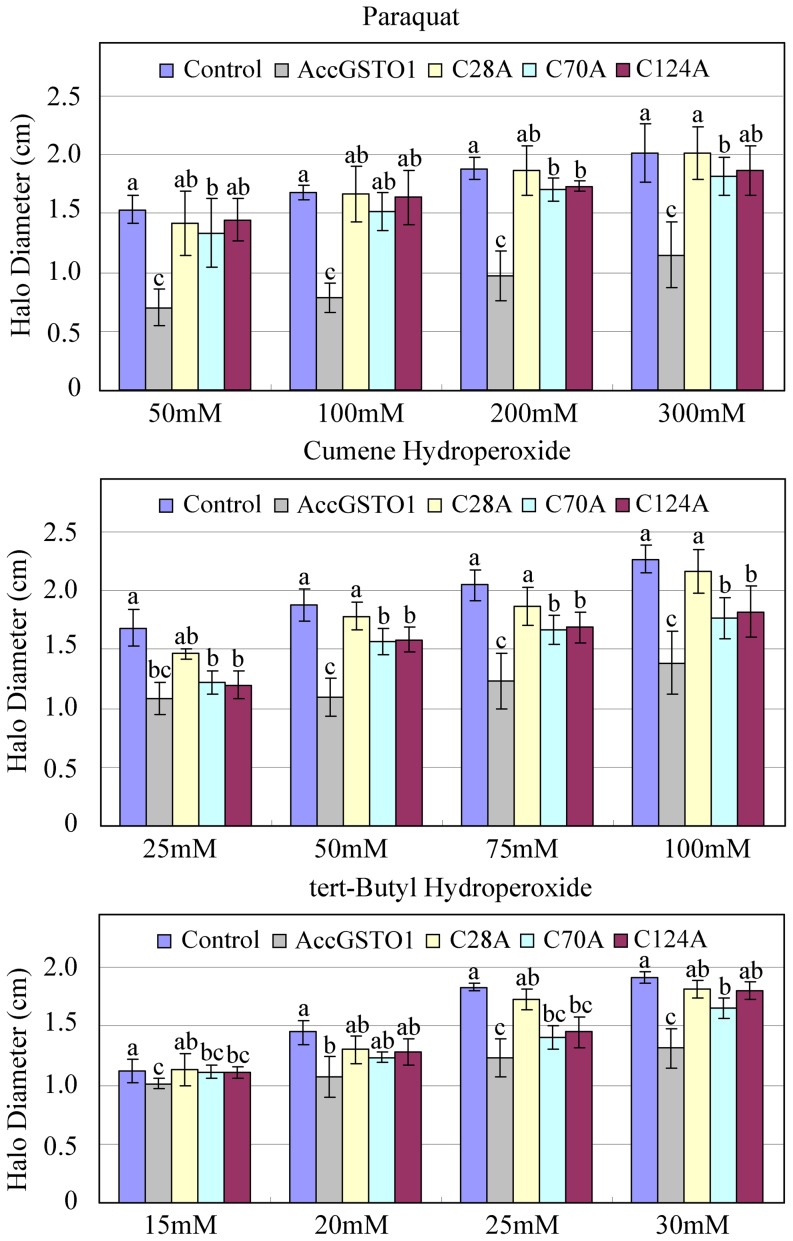
Disc diffusion assays using *E. coli*-overexpressed proteins. The halo diameters of the killing zones were detected after an overnight exposure. LB agar plates were flooded with bacteria overexpressing AccGSTO1, C28A, C70A, or C124A. Bacteria transfected with BL21 (pET-30a (+) only) was used as a control. The data presented are the mean ± S.E.M. of three independent experiments (n = 3). The different letters above the columns indicate significant differences (P<0.05) according to Duncan's multiple range test performed using SAS version 9.1 software.

To evaluate the effect of the three Cys residues on the antioxidant activity of AccGSTO1, *E. coli* overexpressing AccGSTO1, C28A, C70A, and C124A were exposed to oxidative stressors, such as paraquat (internal inducer), cumene hydroperoxide, and tert-butyl hydroperoxide (external inducer). As shown in Figure S2, the ability of AccGSTO1 to protect the *E. coli* cells from oxidative stress was quite obvious. The killing zones were smaller on the plates with the *E. coli* expressing AccGSTO1 than the plates with the controls, with 43–55% (paraquat), 36–42% (cumene hydroperoxide), and 9–33% (tert-butyl hydroperoxide) halo reduction (Fig. 6 and Figure S2). Interestingly, replacing the three Cys residues with Ala resulted in varied losses of antioxidant activity for each residue. Because Cys-28 is present in the active site, the C28A mutant exhibited almost no antioxidant activity when compared to the control bacteria. The alanine mutations of Cys-70 and Cys-124 also decreased the stress response of AccGSTO1, but the antioxidant ability of the C70A or C124A mutants was observed mostly with cumene hydroperoxide treatment.

### Effects of the C28A, C70A, and C124A mutations on DNA damage protection

**Figure 7:**
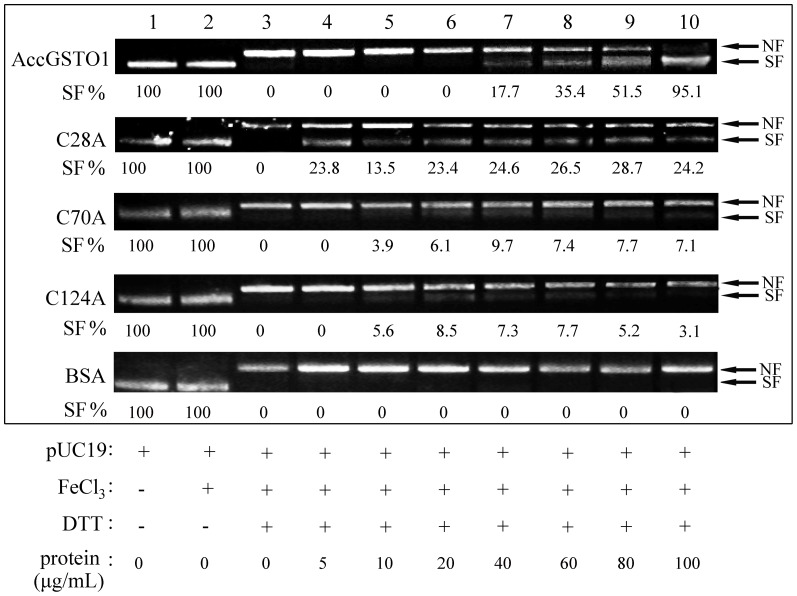
Protection of DNA from oxidative damage by purified proteins in the MFO system. Lane 1, pUC19 plasmid DNA only; lane 2, pUC19 plasmid DNA + FeCl_3_; lane 3, pUC19 plasmid DNA + FeCl_3_ + DTT; lane 4–10, pUC19 plasmid DNA + FeCl_3_ + DTT + purified proteins (5, 10, 20, 40, 60, 80, and 100 μg/mL, respectively). SF, supercoiled form; NF, nicked form. SF% is SF/(SF + NF) for each lane.The expression level was determined by the signal intensities of the bands.

Oxidative stress destroys DNA integrity and blocks specific DNA repair pathways [42], [43]. In the mixed-function oxidation (MFO) system, the electron donor dithiothreitol (DTT) can react with FeCl_3_ to produce hydroxyl radicals, which convert supercoiled DNA to nicked DNA forms [44]. When the pUC19 plasmid was incubated alone or together with FeCl_3_, a completely supercoiled form of DNA was observed; however, when DTT was added, the pUC19 plasmid was nicked, leading to altered migration (Fig. 7). As increasing concentrations of AccGSTO1 were added, the amount of the nicked form gradually decreased, becoming nearly undetectable at 100 μg/mL AccGSTO1 (Fig. 7, lane 10). Notably, regardless of which Cys residue was replaced with Ala, the three mutants were unable to effectively protect the supercoiled DNA from nicking as well as the wild-type AccGSTO1. A control protein (BSA) did not display any nicking protection (Fig. 7). We can conclude that the C28A, C70A, and C124A mutations influence DNA damage protection, which may be conferred by the sulfhydryl moieties of the Cys residues.

### Effects of the C28A, C70A, and C124A mutations on AccGSTO1 enzyme activity

GSTO1 has unique enzymatic properties, such as undetectable CDNB activity and highly specific DHAR activity [22]. AccGSTO1 also exhibited the lowest CDNB activity and the most specific DHAR activity (Table 1). The maximum DHAR activity for AccGSTO1 was observed at pH 8.0 and an optimal temperature of 25°C (Figure S3). At a fixed DHA concentration of 1 mM, the Km and Vmax values for GSH were 1.63±0.41 mM and 4.17±0.87 μmol/min per mg of protein, respectively. At a fixed GSH concentration of 2.25 mM, the Km and Vmax values for DHA were 0.57±0.06 mM and 6.15±1.76 μmol/min per mg of protein, respectively. Additionally, AccGSTO1 exhibited detectable glutathione peroxidase activity against different peroxidase substrates including H_2_O_2_, cumene hydroperoxide or tert-butyl hydroperoxide, which is similar to Gto1 in *Saccharomyces cerevisiae* [45]. However, mutation of AccGSTO1 Cys-28 or Cys-124 caused an increase in CDNB activity and the C28A, C70A, and C124A mutations showed no activity toward DHA, H_2_O_2_, cumene hydroperoxide or tert-butyl hydroperoxide. GSTO1 orthologs display different enzymatic activities across species, and their mutants also show differential losses of activity (Table 1). These results indicate that the Cys residues are important for GSTO1 activities.

**Table 1:** Comparison of specific activities of AccGSTO1 and other GSTO.

	CDNB	DHAR	glutathione peroxidase	cumene hydroperoxide	tert-butyl hydroperoxide	Reference
AccGSTO1	0.015±0.002	1.035±0.041	0.193±0.012	0.081±0.008	0.047±0.003	This study
C28A	0.0225±0.003	ND	ND	ND	ND	This study
C70A	0.0143±0.006	ND	ND	ND	ND	This study
C124A	0.0181±0.003	ND	ND	ND	ND	This study
HsGSTO1	0.18±0.006	0.16±0.005	_	ND	ND	[13]
Gto1	ND	0.23±0.031	ND	ND	ND	[42]
Gto2	ND	0.11±0.009	ND	ND	0.13	[42]
Gto3	ND	0.16±0.004	0.18	0.31	0.99	[42]
bmGSTO	0.67±0.06	0.64±0.08	0.32±0.05	_	_	[24]
C38A	3.8±0.7	ND	ND	_	_	[24]
P39A	ND	ND	ND	_	_	[24]
Y40A	ND	ND	ND	_	_	[24]

Specific activity shown in μmol/min/mg. ND, not detectable. “_”indicated not determined. C38A, P39A and Y40A are mutants for bmGSTO.

### Effects of the C28A, C70A, and C124A mutations on the structure of AccGSTO1

Using Swiss-PdbViewer, we predicted the three-dimensional structures of AccGSTO1 and the C28A, C70A, and C124A mutants based on the only established crystal structure of GSTO1 [13]. All the GSTO1s were composed of 241 amino acids and assembled as a dimer that clearly adopted the canonical GST fold. At the N-terminal domain, a central four-stranded β sheet and two α helices established a thioredoxin-like domain (βαβαββ), whereas larger helices (7α) were found in the C-terminal domain. Notably, of the nine helices, two helices (α2, residues 58–59, and α8, residues 211–214) were 3_10_ helices, and two special β sheets (β5, residues 178–180, and β6, residues 185–187) were found in the C-terminal domain of AccGSTO1 but not in the C28A, C70A, and C124A mutants or in other GST structures.

**Figure 8:**
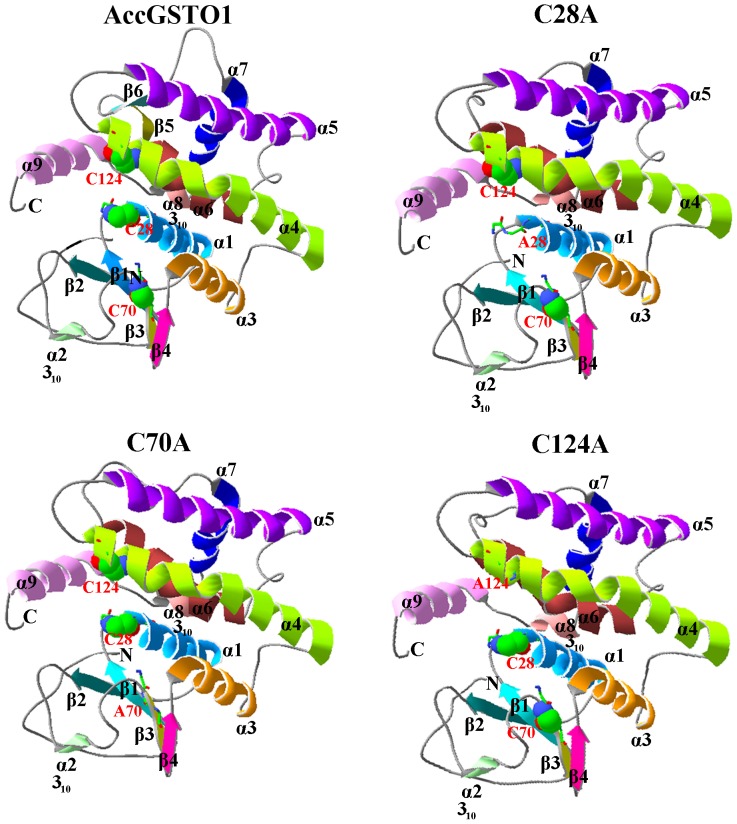
The preidicted tertiary structure of the wild-type AccGSTO1 and C28A, C70A, and C124A mutant proteins. The N-terminus, C-terminus, α-helices, and β sheets are marked. Helices α2 and α8 are 3_10_ helices, and the other helices are α-helices. The Cys and mutagenic Ala residues are highlighted by a ball and stick representation. These images were generated using Swiss-PdbViewer.

**Figure 9:**
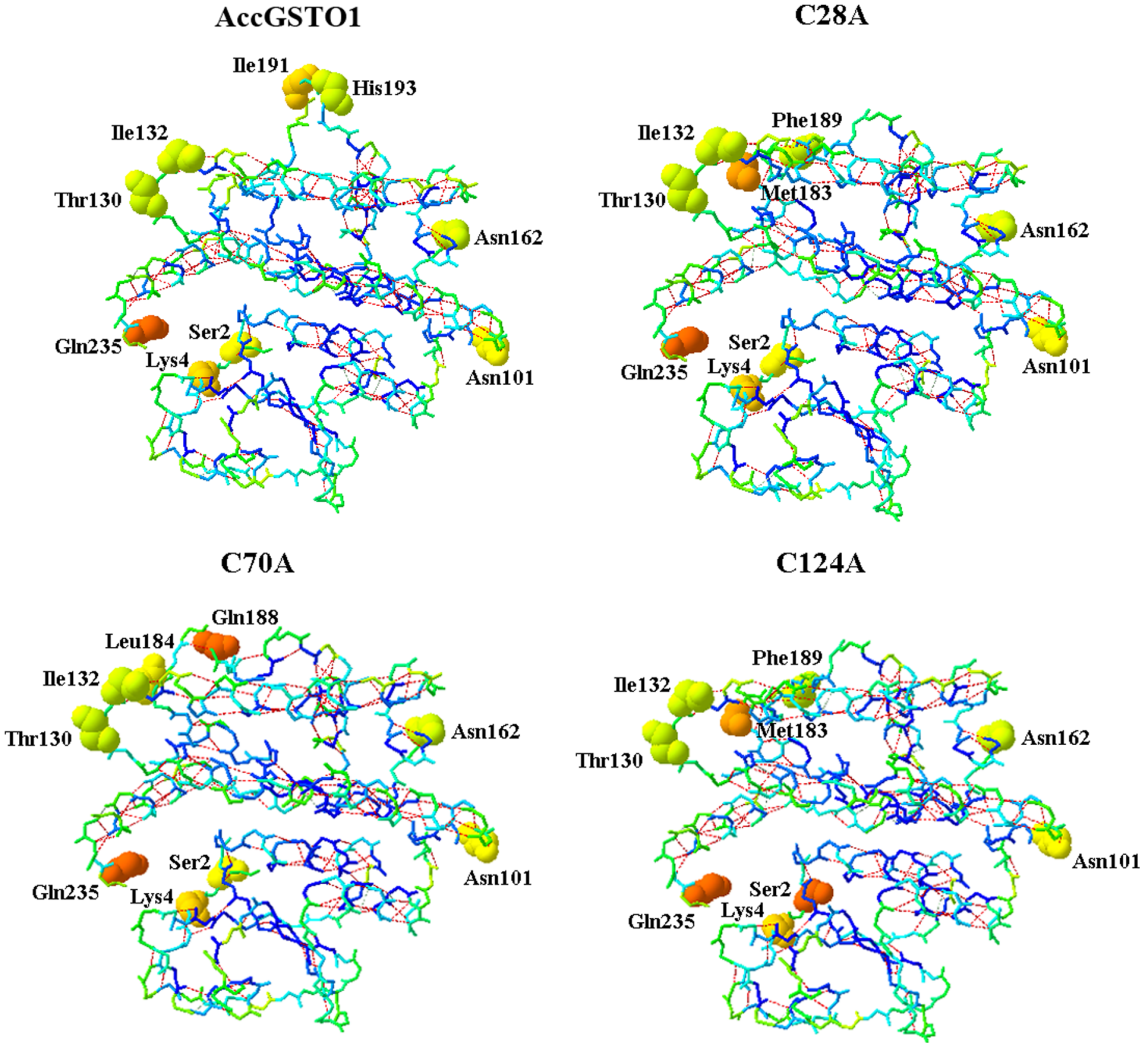
The distribution of H-bonds and different amino acid residues on the surface of the protein. The H-bonds are highlighted in red. The different colors indicate the different locations of the amino acids within the protein. Blue and green represent interior amino acids. The amino acids on the surface of the protein are shown in ball representation. Orange represents the superficial amino acids that were highly contacted by solvent. These images were drawn using Swiss-PdbViewer.

There were some differences in the predicted three-dimensional structures of the three mutants compared with the structure of AccGSTO1 (Fig. 8). For example, large random coils were distributed differently throughout these structures; β5 and β6 in AccGSTO1 were replaced with other secondary structures, including α6 (residues 167–181) and random coils (residues 185–187) in the C28A, C70A, and C124A proteins. These distinct structures were mainly concentrated in the C-terminal domain. As shown in Fig. 9, we found that the H-bonds in the C-terminal domain were markedly altered between AccGSTO1 and the three mutants and among the C28A, C70A, and C124A mutants. Furthermore, the residue solvent accessibilities among these proteins were also changed, such as Ile-191 and His-193 in AccGSTO1, Met-183 and Phe-189 in C28A and C124A, and Leu-184 and Gln-188 in C70A. These predicted structural differences may be the reason for the functional disparities among the wild-type AccGSTO1 and the C28A, C70A, and C124A mutant proteins.

## Discussion

The Omega-class of GSTs (GSTO) is a class of cytosolic GSTs with structural and functional characteristics that differ from those of the other GST groups, such as a specific Cys-32 in the active site, a lower CDNB activity, and unique enzymatic properties (TTase and DHAR) [13], [20], [22], [23]. In this study, we demonstrated the involvement of GSTO1 from *A. cerana cerana* in the oxidative stress response and investigated the influence of different cysteine residues on this response. The first GSTO1 was identified from human expressed sequence tags [13]. Similar to HsGSTO1, the canonical GST structure and unique N-terminal and C-terminal extensions were also found in AccGSTO1. However, three Cys residues were found in AccGSTO1, and the conserved Cys-28 active site residue of AccGSTO1 was not predicted to be located in helix α1. Additionally, two novel β5 and β6 sheets were found in the predicted AccGSTO1 tertiary structure, which is significantly different from the typical GST.

Accumulated evidence suggests that *GSTO1* is involved in antioxidant defense [23], [24], [27], [28]. The highest expression of *GSTO1* was reported in liver and muscle, suggesting that the encoded protein is responsible for the detoxification of xenobiotics and ROS scavenging [13]. The higher tissue expression of *AccGSTO1* was found in the midgut, epidermis, and flight muscle, which differed from the expression of *AccGSTO2* in brain tissue [36]. Although AccGSTO2 may respond to oxidative stress by maintaining ascorbic acid levels in the brain, AccGSTO1 plays an antioxidant role by detoxification in the muscle or midgut. In fact, this distribution pattern of AccGSTO1 is similar to the distribution pattern in vertebrates: honeybees do not have a liver, though the midgut is a highly metabolically active organ that can function in the elimination of toxicants. In addition, the protective role of AccGSTO1 against oxidative stress was also demonstrated in different developmental stages. Foragers are the major pollinators of flowering plants and are often exposed to a variety of environmental stimuli, such as temperature, UV light, and pesticides. As the autologous enzymatic antioxidant systems are more powerful in younger individuals than older individuals [8], it was not surprising that the lowest expression of *AccGSTO1* was detected in dead bees. In humans, several reports have confirmed a close association between *GSTO1* and age-related diseases [6], [7]. In honeybees, the specific silencing of the *AccGSTO1* gene using RNAi notably decreased their survival rate at high temperature, which was related to higher oxidative stress. Our findings may provide valuable insight into the correlation between life span and antioxidant activity.

The direct evidence of an antioxidant role was derived from the experiments that examined responses to cold and heat shock, resistance to peroxides, and DNA damage protection. Temperature is one of the dominant abiotic factors directly affecting herbivorous insects, and the optimal temperature of honeybee colonies is between 33°C and 36°C [46]. Low temperatures decrease metabolic rates and increase oxygen radical formation during flight [47]; conversely, heat stress accelerates mitochondrial respiration and also stimulates the formation of ROS [48]. When honeybees are subjected to cold or heat stress, lipid peroxides begin to accumulate faster, resulting in higher MDA concentrations. The expression of *AccGSTO1* was significantly induced at 4°C, 15°C, and 43°C in a short time period, specifically by 1 h, suggesting that *AccGSTO1* perhaps mediates an immediate antioxidant activity when excessive ROS are generated. While oxidative stress was beyond the scavenging rate of autologous enzymatic antioxidant systems, *AccGSTO1* expression was decreased. Furthermore, the RNAi-mediated silencing of *AccGSTO1* in foragers increased their sensitivity to heat shock and raised MDA levels. Therefore, when organisms suffer long-term injuries or become older, the antioxidant functions sharply decrease or disappear.

In addition to temperature, cumene hydroperoxide and tert-butyl hydroperoxide can also act as external environmental stressors. Paraquat can act as an intracellular ROS inducer by crossing cell membranes to generate the oxygen radical superoxide anion (O_2_^–^) during aerobic metabolism [23]. The disc diffusion assay clearly showed that *AccGSTO1* protected against oxidative stress, with the intracellular inducer-mediated oxidative stress being more significantly inhibited than the externally induced oxidative stress. In contrast, the resistance to cumene hydroperoxide and tert-butyl hydroperoxide in *C. elegans* increased 3-fold compared to paraquat resistance [23]. This discrepancy may be due to species differences or the specific structures of AccGSTO1 that are distinct from classic GSTO1. Of the many biological targets of oxidative stress, nucleic acids are often affected [42]; indeed, ROS can inhibit DNA damage repair and uncouple oxidative phosphorylation [42], [43]. In the MFO system, the C28A mutant protected a small amount of supercoiled plasmid at the low concentration, indicating that it retained some antioxidant activity. We speculated that each Cys residue plays a different role in the response to oxidative stress. Cys28 is at the active site and may be required for AccGSTO1 function. Moreover, regardless of which Cys residue was replaced with Ala, the three mutants were unable to effectively protect the supercoiled form of plasmid DNA as well as the wild-type AccGSTO1, especially at protein concentrations greater than 60 μg/mL. AccGSTO1 protected DNA integrity and was involved in DNA damage protection, which is similar to other antioxidant enzymes such as thioredoxin peroxidase [49]. These results demonstrate that AccGSTO1 plays a key role in counteracting oxidative stress.

This protective role against oxidative stress is mediated by glutathione (GSH), which can neutralize a component of toxic xenobiotics. Similar to other GSTs, GSTO1 has a well-defined active site that is adjacent to the GSH-binding site. The H-site is constructed from elements of both the N-terminal and C-terminal domains [13]. However, GSTO1 has unique N-terminal and C-terminal extensions that are not found in other GST classes, and these variations reflect its varied substrate specificity [21]. Although some residues are conservatively replaced in AccGSTO1, these residues also form the walls of the pocket that corresponds to the H-site in the HsGSTO1 structure [13]. The Phe-27 and Pro-29 residues in AccGSTO1 form a wide, deep pocket, and Arg-177 contributes to the bottom of the pocket; residues in the C-terminal α9 helix, particularly His-222, form the top and back of the pocket. The structural similarity suggests that AccGSTO1 has the same substrate specificity as HsGSTO1. In this study, AccGSTO1 exhibited the low CDNB activity, glutathione peroxidase activity and specific DHAR activity, which are similar characteristics to the majority of GSTO1 proteins [13], [22], [23], [24], [43].

Unlike HsGSTO1, there are three Cys residues in AccGSTO1. Although the active site residue Cys-28 in AccGSTO1 is not located in helix α1, it has been demonstrated that this Cys residue is crucial for forming a mixed disulphide bond with GSH [50], [51]. Mutations of some other important residues have also been reported. For example, a Cys-32 to Ala mutation in human *GSTO1* strongly increases activity toward CDNB [22], whereas mutations at Cys-38 and Pro-39 in silkmoths lead to largely negative changes in the activities of BmGSTO1 [24], and an Asp-140 variant has decreased transferase activity [32]. In the present study, three mutants, C28A, C70A, and C124A, exhibited no enzymatic activity against DHA. Interestingly, the Cys-28 and Cys-124 mutations in AccGSTO1 caused an increase in activity toward CDNB, though C70A had no influence on CDNB activity. An interesting result was also observed in the disc diffusion assay: the C28A protein had almost no antioxidant activity, whereas the antioxidant activity was retained in the C70A and C124A mutants mostly with cumene hydroperoxide. These results may be ascribed to variations in the GSTO1 structure. Cys-28 is located in the active site in the N-terminal domain and Cys-124 is located in the α4 helix in the C-terminal region, which can form a distinct structural unit by attachment of the N-terminal extension [13]. The predicted tertiary structure of the C28A and C124A proteins displayed dramatic alterations in their C-terminal domains compared with AccGSTO1; in particular, the β5 and β6 sheets of AccGSTO1 were replaced with the α6 helix and a random coil. Interestingly, many different random coils were predicted among the three mutants, and the C70A mutant had more differences than the C28A and C124A mutants. Hydrogen bonds play an important role in maintaining protein stability [52], and these distinct structures influenced the location of several H-bonds. Furthermore, the change in protein structure also affected the residue solvent accessibilities that related to hydrophobic interactions. For example, Ile-191 and His-193 in AccGSTO1 were internalized in the three mutants, but Met-183 and Phe-189 became surface residues in C28A and C124A. Moreover, Leu-184 and Gln-188 of C70A had more opportunity to bind to solvents. Cys-replacement mutants changed their solvent accessibilities and hydrophobic interactions, which are the major driving forces for protein structure [53].

Protection against oxidative stress is an eternal theme for the life span of living organisms. In conclusion, the *GSTO1* gene from *A. cerana cerana* is involved in the response to oxidative stress, and three Cys residues play pivotal roles in its antioxidant activity, particularly Cys-28 and Cys-124. These findings provide valuable insight into the functions of Omega-class GSTs in the oxidative stress response.

## Supporting Information

Figure S1 **SDS-PAGE analysis of recombinant proteins with a His-tag.** (A) Lane 1: total cellular extract from uninduced *E. coli* BL21 (DE3) cells (WT). Lanes 2, 3, 4, and 5: total cellular extract from induced overexpression of pET-30a (+)-AccGSTO1, pET-30a (+)-C28A, pET-30a (+)-C70A, and pET-30a (+)-C124A, respectively. (B) Lanes 1, 2, 3, and 4: purified recombinant AccGSTO1, C28A, C70A, and C124A proteins, respectively. M: low molecular weight protein marker. Target proteins are indicated by arrows.
(TIF)

Figure S2 **Disc diffusion assays using** ***E. coli*** **-overexpressed proteins.** (A) The filter discs were soaked in different concentrations of paraquat (filter discs 2, 3, 4 and 5: 50, 100, 200 and 300 mM, respectively), cumene hydroperoxide (filter discs 2, 3, 4 and 5: 25, 50, 75 and 100 mM, respectively), or tert-butyl hydroperoxide (filter discs 2, 3, 4 and 5: 15, 20, 25 and 30 mM, respectively). Filter discs 1 were soaked with water. The discs were placed on the agar plates, which were incubated overnight; the killing zones around the oxidant-soaked filters were then measured. (B) Decreased percentages of killing zones for AccGSTO1 compared with controls under different oxidant concentrations.
(TIF)

Figure S3 **The effects of pH (A) and temperature (B) on the DHAR activity of AccGSTO1.** Different pHs (4.0–9.0) and temperatures (10–50°C) were selected to determine the optimal pH and temperature.
(TIF)

Table S1 **Primer information in this study.**
(DOC)
