# Supplementary figures and images for: Characterization and Mutational Analysis of Omega-Class GST (GSTO1) from Apis cerana cerana, a Gene Involved in Response to Oxidative Stress
Source: PLoS One. 2014 Mar 25;9(3):e93100. doi: 10.1371/journal.pone.0093100 (PMC3965517; doi:10.1371/journal.pone.0093100)

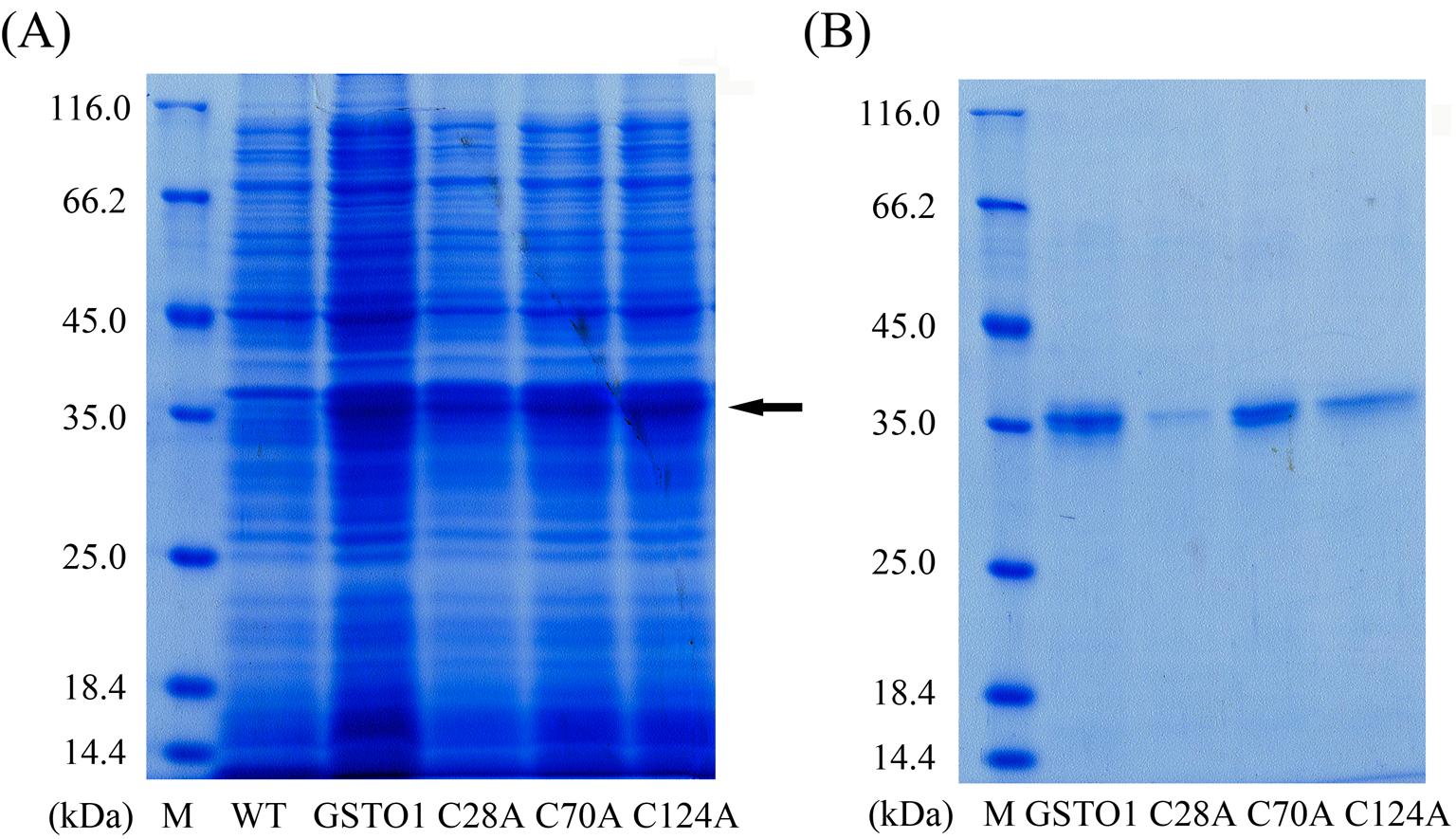

Supplement: Figure S1 — SDS-PAGE analysis of recombinant proteins with a His-tag. (A) Lane 1: total cellular extract from uninduced E. coli BL21 (DE3) cells (WT). Lanes 2, 3, 4, and 5: total cellular extract from induced overexpression of pET-30a (+)-AccGSTO1, pET-30a (+)-C28A, pET-30a (+)-C70A, and pET-30a (+)-C124A, respectively. (B) Lanes 1, 2, 3, and 4: purified recombinant AccGSTO1, C28A, C70A, and C124A proteins, respectively. M: low molecular weight protein marker. Target proteins are indicated by arrows. (TIF) [file pone.0093100.s001.tif]

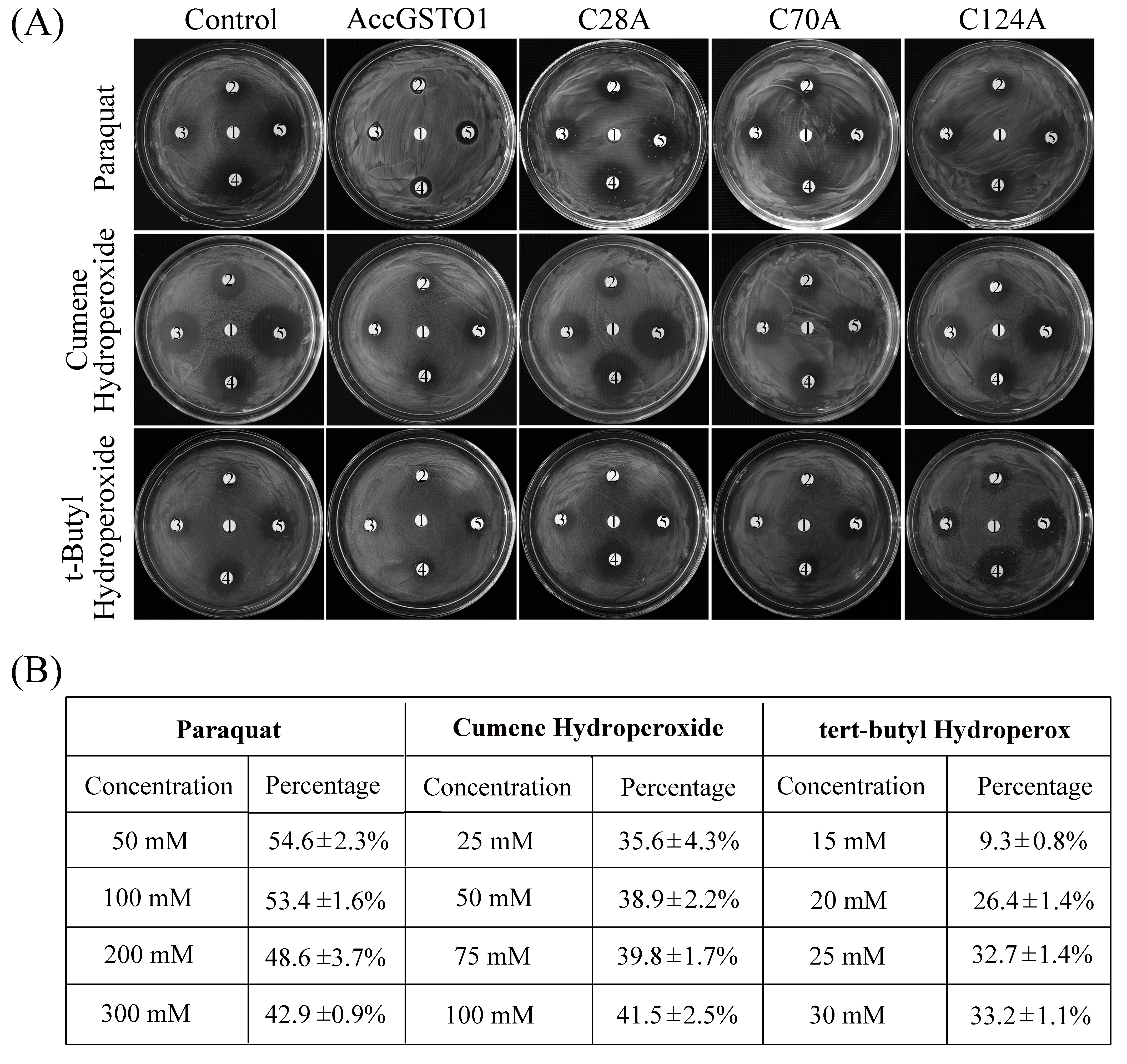

Supplement: Figure S2 — Disc diffusion assays using E. coli -overexpressed proteins. (A) The filter discs were soaked in different concentrations of paraquat (filter discs 2, 3, 4 and 5: 50, 100, 200 and 300 mM, respectively), cumene hydroperoxide (filter discs 2, 3, 4 and 5: 25, 50, 75 and 100 mM, respectively), or tert-butyl hydroperoxide (filter discs 2, 3, 4 and 5: 15, 20, 25 and 30 mM, respectively). Filter discs 1 were soaked with water. The discs were placed on the agar plates, which were incubated overnight; the killing zones around the oxidant-soaked filters were then measured. (B) Decreased percentages of killing zones for AccGSTO1 compared with controls under different oxidant concentrations. (TIF) [file pone.0093100.s002.tif]

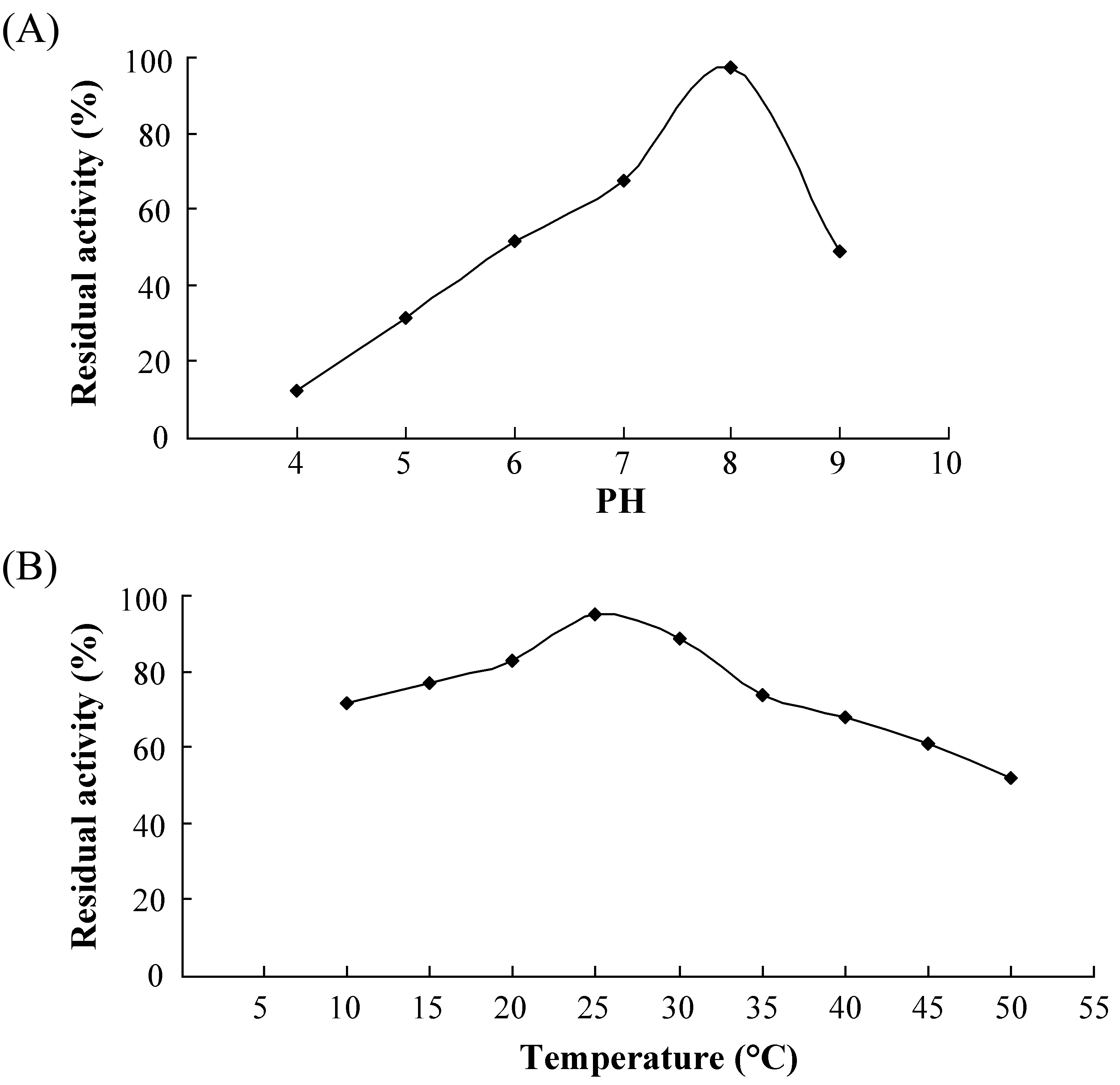

Supplement: Figure S3 — The effects of pH (A) and temperature (B) on the DHAR activity of AccGSTO1. Different pHs (4.0–9.0) and temperatures (10–50°C) were selected to determine the optimal pH and temperature. (TIF) [file pone.0093100.s003.tif]
