# Supplementary material for: Characterization and Mutational Analysis of Omega-Class GST (GSTO1) from Apis cerana cerana, a Gene Involved in Response to Oxidative Stress
Source: PLoS One. 2014 Mar 25;9(3):e93100. doi: 10.1371/journal.pone.0093100 (PMC3965517; doi:10.1371/journal.pone.0093100)
Supplement: Table S1 — Primer information in this study. (DOC) [file pone.0093100.s004.doc]

**Table S1.** Primer information in this study

| **Primer** | **Primer sequence (5'→3')** |
| --- | --- |
| Internal fragment primers |  |
| GP1 | ATGAGTTCTAAACATTTGACTATCG |
| GP2 | TTAATCAGTAATCAAATCATATTGTGG |
| 5'-RACE primers |  |
| 5P1 | GAGGATCATTAGGATATAGTTTG |
| 5P2 | CCGATAGTCAAATGTTTAGAACTCAT |
| AAP | GGCCACGCGTCGACTAGTACG(G)_14_ |
| AUAP | GGCCACGCGTCGACTAGTACG |
| 3'-RACE primers |  |
| 3P1 | GTTCCATGTATAGAATTAGAAGGAGGAG |
| 3P2 | CATTCTTTCATGGTAATTCTCCTGGC |
| B26 | GACTCGAGTCGACATCGA(T)_18_ |
| B25 | GACTCGAGTCGACATCGA |
| The full-length cDNA primers |  |
| Q1 | ATGAGTTCTAAACATTTGACTATCG |
| Q2 | TTAATCAGTAATCAAATCATATTGTGG |
| The cloning of the genomic sequence |  |
| QG1 | GTCAAGTGTAAATCTAACCTTCAG |
| QG2 | TTAATCAGTAATCAAATCATATTGTGG |
| Primers used in prokaryotic expression |  |
| Y1 | GGTACCATGAGTTCTAAACATTTGACTATCG |
| Y2 | GAGCTCATCAGTAATCAAATCATATTGTGG |
| Site-directed mutagenesis |  |
| TBS1 | TATAGTATGCGATTTGCTCCATATGCCCAAAG |
| TBX1 | GCAAATCGCATACTATATAAACGTATTTTTCC |
| TBS2 | TTGGGTAAAGTTCCAGCTATAGAATTAGAAGG |
| TBX2 | GCTGGAACTTTACCCAAAGGACTTTTTTCTAA |
| TBS3 | GTTATAAATACTATGGCTAAGTTGTTTATTAA |
| TBX3 | GCCATAGTATTTATAACAGAATTAAATCTACC |
| Primers used in RNAi |  |
| DR1 | TAATACGACTCACTATAGGGCGAATGAGTTCTAAACATTTGACTATCG |
| DR2 | TAATACGACTCACTATAGGGCGAGAGGATCATTAGGATATAGTTTG |
| dsGFP1 | TAATACGACTCACTATAGGGCGAAGTGGAGAGGGTGAAG |
| dsGFP2 | TAATACGACTCACTATAGGGCGAGGTAAAAGGACAGGGC |
| Real-time PCR primers |  |
| YG1 | CATTCTTTCATGGTAATTCTCCTGGC |
| YG2 | TTAATCAGTAATCAAATCATATTGTGG |
| β1 | GTTTTCCCATCTATCGTCGG |
| β2 | TTTTCTCCATATCATCCCAG |
